# The Hsp90–Sti1 interaction is critical for *Leishmania donovani* proliferation in both life cycle stages

**DOI:** 10.1111/cmi.12057

**Published:** 2012-11-20

**Authors:** Antje Hombach, Gabi Ommen, Mareike Chrobak, Joachim Clos

**Affiliations:** Bernhard Nocht Institute for Tropical MedicineHamburg, Germany

## Abstract

The heat shock protein 90 plays a pivotal role in the life cycle control of *Leishmania donovani* promoting the fast-growing insect stage of this parasite. Equally important for insect stage growth is the co-chaperone Sti1. We show that replacement of *Sti1* is only feasible in the presence of additional *Sti1* transgenes indicating an essential role. To better understand the impact of Sti1 and its interaction with Hsp90, we performed a mutational analysis of Hsp90. We established that a single amino acid exchange in the *Leishmania* Hsp90 renders that protein resistant to the inhibitor radicicol (RAD), yet does not interfere with its functionality. Based on this RAD-resistant Hsp90, we established a combined chemical knockout/gene complementation (CKC) approach. We can show that Hsp90 function is required in both insect and mammalian life stages and that the Sti1-binding motif of Hsp90 is crucial for proliferation of insect and mammalian stages of the parasite. The Sti1-binding motif in *Leishmania* Hsp90 is suboptimal – optimizing the motif increased initial intracellular proliferation underscoring the importance of the Hsp90–Sti1 interaction for this important parasitic protozoan. The CKC strategy we developed will allow the future analysis of more Hsp90 domains and motifs in parasite viability and infectivity.

## Introduction

Heat shock proteins (Hsps), also termed chaperones, comprise several highly conserved families of protein folding facilitators that are important in every aspect of cell functionality. Among the Hsps, the 90 kDa family (Hsp90) was shown to be of particular impact on aspects such as gene regulation, signal transduction, tumour suppression and the suppression of mutation-induced phenotypes (Rutherford and Zuker, 1994; Rutherford and Lindquist, 1998; Queitsch *et al*., 2002). The latter function gives Hsp90 an important role in evolution.

The interaction of Hsp90 with steroid hormone receptors was unravelled first (Sanchez *et al*., 1987; Bresnick *et al*., 1989; Pratt *et al*., 1989), but soon it became clear that a large group of regulatory protein factors depend on Hsp90 for their function (Rutherford and Zuker, 1994), including the heat shock transcription factors of metazoa (Ali *et al*., 1998). Since then, the participation of Hsp90 in other control functions became known, including involvements in tubulin assembly/disassembly, antigen presentation and resistance to toxic materials (Morimoto *et al*., 1990; Young, 1992; Kaufmann and Schoel, 1994, Minowada and Welch, 1995; Smith *et al*., 1998; Vergnes *et al*., 2007).

Much was learned about the true range of Hsp90 functions by the introduction of Hsp90-specific inhibitors (Schulte *et al*., 1998; Sharma *et al*., 1998; Smith *et al*., 1998). Both geldanamycin and radicicol exert their inhibitory effects through their high affinity for the Hsp90 ATP-binding pocket (Roe *et al*., 1999). This chemical knockout approach also led to the establishment of Hsp90 inhibitors as prospective cancer therapeutics (Whitesell and Lindquist, 2005).

The multitude of functions is reflected in the varieties of Hsp90-containing chaperone complexes. Hsp90 always forms the dimeric core of such ‘foldosome’ complexes and it associates with other chaperones such as Hsp70 and Hsp40 family members, but also with a variety of so-called co-chaperones (Scheibel and Buchner, 1998; Buchner, 1999; Wandinger *et al*., 2008).

Of the latter, the stress-inducible protein Sti1 is most widely conserved being found in organisms as diverse as kinetoplastid protozoa and mammals (Johnson and Brown, 2009). Sti1 possesses three Hsp interaction domains, each consisting of three so-called tetratricopeptide repeat (TPR) motifs, which recognize and bind to the cognate recognition motifs on Hsp90 and Hsp70, C′-terminal MEEVD-C′ or VEEVD-C′ motifs respectively. Recent evidence suggests that only one Hsp90 moiety is bound by Sti1 at any given point in time; the other Hsp90 may interact with a different co-chaperone (Li *et al*., 2011).

In *Leishmania*, Hsp90 was first identified as a highly abundant and immunogenic molecule. Hsp90, also known as Hsp83 in *Leishmania* spp, is encoded by up to 17 identical, tandemly arranged gene copies per haploid set of chromosomes (Hubel and Clos, 1996; Zilka *et al*., 2001), and it accounts for almost 3% of the extractable protein fraction (Brandau *et al*., 1995). Its interaction with at least one co-chaperone, Sti1 or HOP (Hsp organizing protein), was also shown early on (Webb *et al*., 1997).

Contrary to early speculation, most heat shock proteins of *Leishmania* only show a transient and limited induction (Brandau *et al*., 1995; Rosenzweig *et al*., 2008) when the parasites transform from the insect-dwelling, flagellated promastigote stage into the primarily intracellular, non-flagellated amastigote. The latter stage is pathogenic to mammals. This differentiation is triggered in large part by the higher temperatures in mammalian tissues and the acidic environment of the phagosomes in the host's macrophages. Indeed, a similar temperature and pH *in vitro* can trigger an extracellular conversion of promastigotes to so-called axenic amastigotes, an *in vitro* stage that is frequently used experimentally instead of intracellular amastigotes (Zilberstein and Shapira, 1994; Krobitsch *et al*., 1998; Saar *et al*., 1998; Barak *et al*., 2005; Rosenzweig *et al*., 2008).

Interestingly, the pharmacological inactivation of Hsp90 using inhibitors such as geldanamycin (GA) or radicicol (RAD) not only leads to cell cycle arrest and the induction of heat shock protein synthesis, but also to a conversion towards an amastigote-like morphology (Wiesgigl and Clos, 2001). This indicated that Hsp90 promotes the promastigote stage, and that the higher temperatures of the mammalian host somehow interfere with that role, thus causing amastigote differentiation.

Recently, Hsp90 and Sti1 were also identified as targets for amastigote stage-specific phosphorylation, linking signal transduction cascades and the chaperone system as likely key actors in life cycle control (Morales *et al*., 2010). That, and the essential roles of some *Leishmania* co-chaperones (Morales *et al*., 2010; Ommen *et al*., 2010), led us to explore the interaction of Hsp90 with Sti1.

This was impeded by the high number of tandemly arranged Hsp90 gene copies found in *Leishmania*. Up to 18, presumably identical copies are found on chromosome 33 (Hubel and Clos, 1996; Zilka *et al*., 2001; Folgueira and Requena, 2007; Ommen and Clos, 2009) extending over > 60 kb and making gene replacement attempts unlikely to succeed. There remained the option of using a pharmacological knockout of the endogenous Hsp90 complemented by a drug-resistant variant. The *Caenorhabditis elegans* Hsp90 was reported to be GA resistant but the structural basis for the low affinity of the inhibitor is unknown. Moreover, the *C. elegans* Hsp90 could not confer GA resistance to another species, presumably because of its inability to interact with co-chaperones or client proteins there (David *et al*., 2003).

Another finding however, the discovery of a single-amino-acid variation in the Hsp90 of *Humicola fuscoatra* that renders this chaperone RAD-resistant, was more promising. An equivalent leucine-to-isoleucine exchange in the *Saccharomyces cerevisiae* Hsp90 rendered protein and organism RAD-resistant (Prodromou *et al*., 2009). In this work, we established a combined chemical knockout/genetic complementation (CKC) approach, using episomal, modified Hsp90 transgenes under RAD pressure. The aim of this study was to analyse the Hsp90–Sti1 interaction in *Leishmania* and to establish its impact on viability and infectivity of *Leishmania donovani* promastigotes and amastigotes.

## Results

### Sti1 gene replacement analysis

Earlier work done in our laboratory indicated a crucial role for LdSti1 for the proliferation of *L. donovani* (Morales *et al*., 2010). Here, we confirm this by using the formal approach, targetting the two Sti1 alleles of *L. donovani* sequentially with gene replacement constructs (Fig. S1A). Monoallelic replacement could be achieved (Fig. S2B, lane 1 and 2). Repeated attempts to replace the second allele yielded four clones that showed dual antibiotic resistance. However, all four still tested positive for Sti1-coding sequences in a PCR assay (Fig. S2A, lanes 1–4). To establish the functionality of the replacement constructs, we attempted the second allele Sti1 gene replacement after integrating a functional Sti1 transgene (pIR-Sti1, Fig. S1B) in the small subunit rRNA gene. Triple antibiotic-resistant promastigotes could be selected.

We tested the gDNA from six candidate clones by PCR amplification of the gene locus (Fig. S2B) expecting the loss of the 3795 bp band representing the intact Sti1 gene locus (lane 1) and the appearance of two PCR products of 2754 bp (puroAC replacement) and 2541 (BleoR replacement). While the monoallelic replacement clone (lane 2) showed the expected two bands at 3795 bp and 2754 bp, we observed a complete lack of the 3795 bp PCR product for all double-allelic replacement candidates (lanes 3–8). Therefore, complete replacement of the natural alleles could be achieved in the presence of a functional transgene. This is strong evidence for an essential role played by Sti1 in the cultured promastigotes and confirms earlier, more preliminary findings.

### Colocalization studies of Sti1 and two major Hsps

**Fig. 1:**
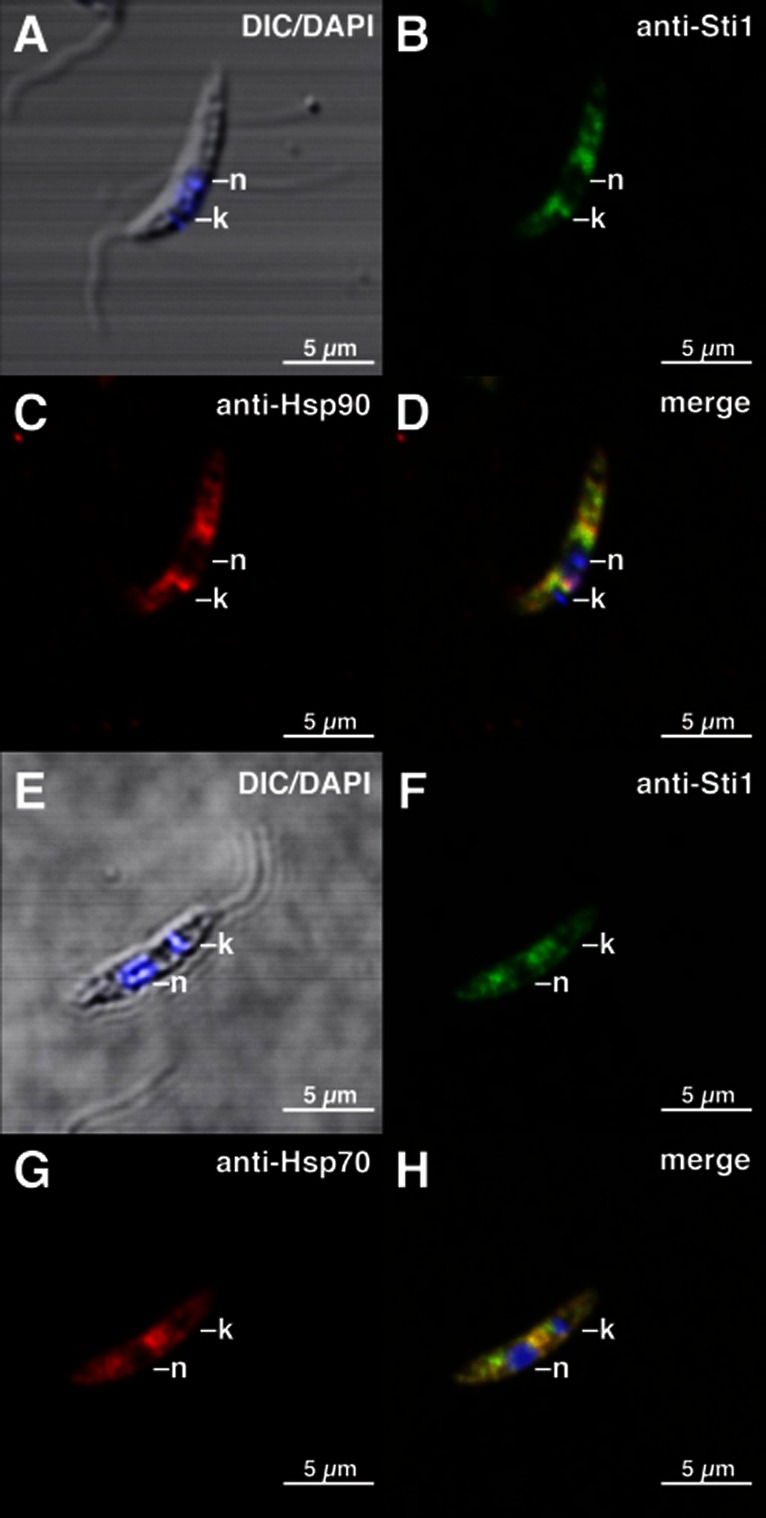
Confocal laser microscopy of *L. donovani*. Parasites were co-stained with DAPI, with mouse anti-Sti1 serum and with chicken anti-Hsp90 and anti-Hsp70 respectively. (A, E) Merge of DIC imaging and DAPI staining; (B, F) Sti1 distribution; (C) Hsp90 distribution; (D) merge of DAPI, anti-Sti1 and anti-Hsp90; (G) Hsp70 distribution; (H) merge of DAPI, anti-Sti1 and anti-Hsp70. The size bars represent 5 μm. k, kinetoplast; n, nucleus.

It was shown by co-immunoprecipitation analysis (Webb *et al*., 1997) that *L. major* Sti1 binds to Hsp90 (a.k.a. Hsp83) and Hsp70. To assess the degree of mutual association, we performed a subcellular localization analysis using specific antibodies and indirect immune fluorescence. Figure 1 shows representative images taken by confocal laser microscopy. Both Sti1 and Hsp90 show a cytoplasmatic distribution with increased signal strength surrounding the nucleus (Fig. 1B and C). Merging the stains clearly shows a stringent colocalization of both proteins (Fig. 1D). A similar colocalization is observed with Sti1 and Hsp70 (Fig. 1E–H). We conclude that Sti1 colocalizes both with Hsp90 and with Hsp70, corroborating its putative role as Hsp organizing protein.

### Evaluation of a RAD-resistant Hsp90rr variant

**Fig. 2:**
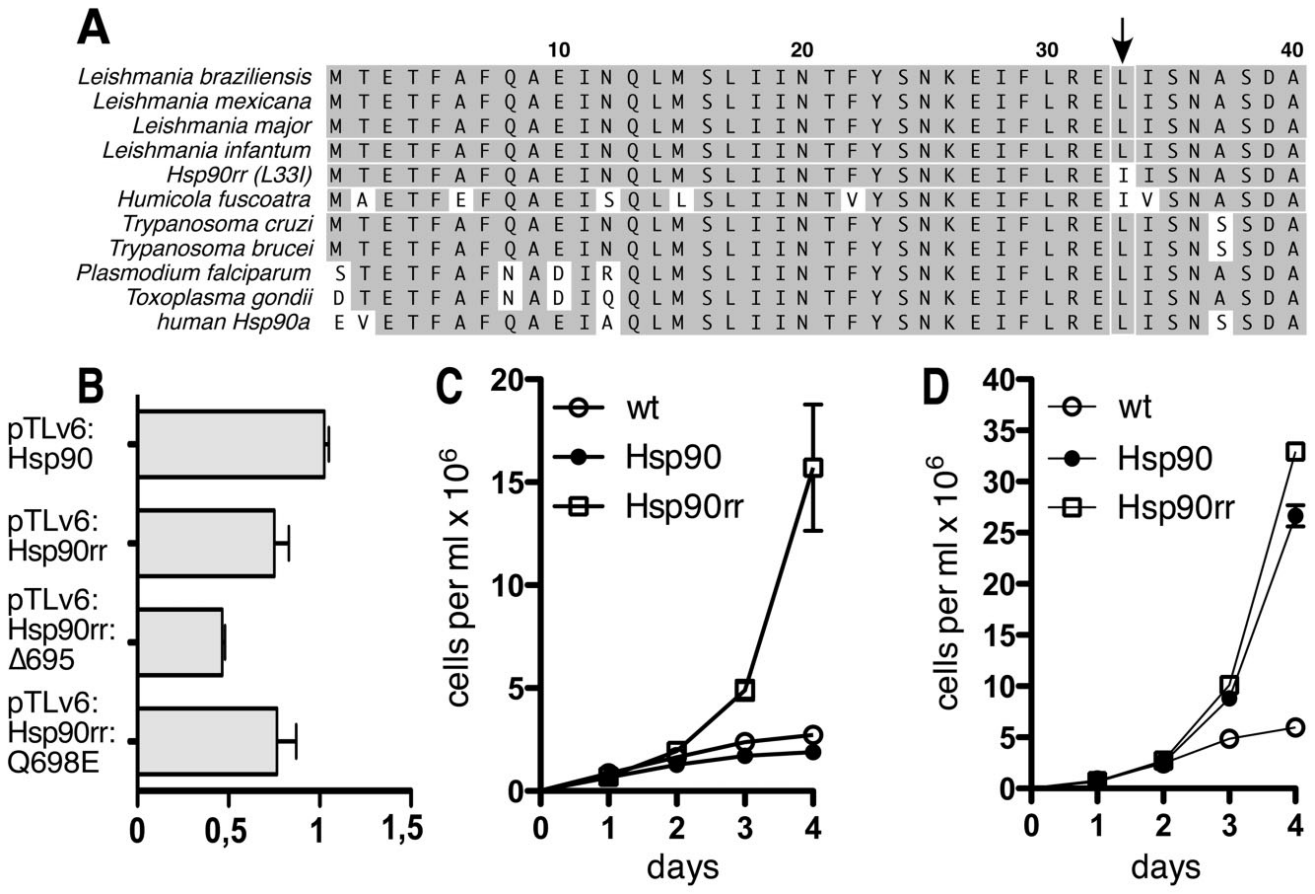
A. Alignment of the N′-terminal 40 amino acids of *Leishmania* spp. Hsp90s with the orthologous sequences of Hsp90s from other species. The arrow points at leucine 33 which is changed to isoleucine in *Humicola fuscoatra* Hsp90 and in the Hsp90rr variant of *Leishmania donovani* Hsp90. B. Semi-quantitative analysis of Hsp90 expression from pTLv6:Hsp90, pTLv6:Hsp90rr, pTLv6:Hsp90rr:Δ695 and pTLv6:Hsp90:Q698E. The selected primers can only amplify cDNA derived from the transgenes. C. *In vitro* proliferation of promastigotes under 5 ng ml^−1^ RAD. Strains *L. donovani* 1SR (open circles), *L. donovani* [pTLv6:Hsp90] (solid circles) and *L. donovani* [pTLv6:Hsp90rr] (open squares) were seeded at 1 × 10^5^ ml^−1^ and grown at 25°C for 4 days. Cell density is plotted against time. D. As in (C), but growth was monitored under 3.75 ng ml^−1^ GA.

Like Hsp90, Hsp70 is encoded by a tandem gene cluster (Lee *et al*., 1988; Bock and Langer, 1993; Quijada *et al*., 1997) and no known specific inhibitors exist. We therefore decided to focus on the interaction of Sti1 with Hsp90. To this end, we needed to establish a system for testing the phenotype of mutated Hsp90. We first evaluated the impact of a L33I amino acid exchange (Fig. 2A) in the *L. donovani* Hsp90 on the parasite's sensitivity to RAD-mediated growth inhibition. Reasoning that a strong expression of the modified Hsp90rr gene was required to compensate for the inactivation of the highly abundant Hsp90, we constructed the episomal expression vector pTLv6 first using pUC19 (Yanisch-Perron *et al*., 1985) cut with SacI and SalI, the 3515 bp SacI–SalI fragment of pcosTL (Kelly *et al*., 1994), and the BamHI fragment (residues 268–2514) of pIRmcs3+ (Hoyer *et al*., 2004). The new vector, pTLv6, was then used to integrate the natural Hsp90-coding sequence (pTLv6:Hsp90) and the presumed, RAD-resistant L33I variant (pTLv6:Hsp90rr). The plasmid maps are shown in Fig. S3. The former construct, pTLv6:Hsp90 was used as control to exclude gene dosis effects due to elevated Hsp90 expression.

After transfection, the episomes were stabilized under G418 selection, and the mRNA levels from Hsp90 and Hsp90rr transgenes were compared by qPCR. Both Hsp90 transgenes were expressed at comparable levels (Fig. 2B). This established, we determined the inhibiting concentration 90 (IC_90_) for RAD on *L. donovani* 1SR promastigotes and performed growth kinetics for *L. donovani* (LdWT), *L. donovani* [pTLv6:Hsp90] and *L. donovani* [pTLv6:Hsp90rr]. As shown in Fig. 2C, *L. donovani* cells expressing the Hsp90rr transgene show growth under RAD IC_90_. Neither wild-type *L. donovani* nor the cells overexpressing the natural Hsp90-coding sequence show proliferation. This is proof that growth under RAD is wholly dependent on the expression of a Hsp90rr variant carrying the crucial L33I exchange. In addition, the sequence variant does not interfere with Hsp90 functionality as evidenced by the unimpeded growth of the Hsp90rr parasites under RAD.

We also tested the potential of the Hsp90rr variant to overcome GA-induced growth inhibition. We observe protective effects both with Hsp90 overexpression and with Hsp90rr (Fig. 2D), indicating a gene dosis-dependent titration effect of Hsp90 overexpression similar to the one observed in earlier studies (Wiesgigl and Clos, 2001).

### Hsp90rr abrogates morphogenic effects of RAD

**Fig. 3:**
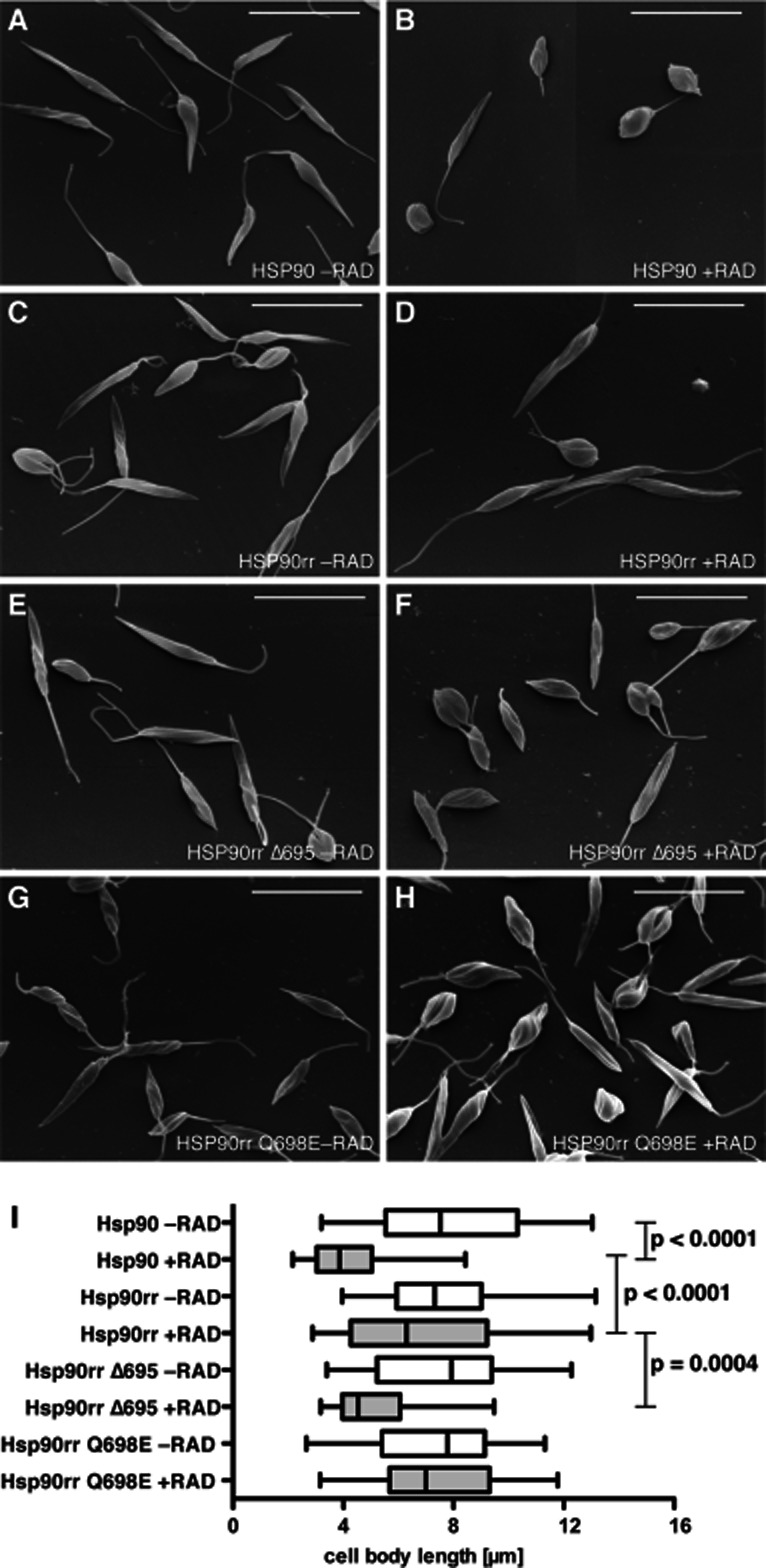
Scanning electron microscopic (SEM) analysis of recombinant *L. donovani* populations. A–H. Promastigotes transfected with pTLv6:Hsp90 (A, B), pTLv6:Hsp90rr (C, D), pTLv6:Hsp90rr:Δ695 (E, F) and pTLv6:Hsp90rr:Q698E (G, H) were cultured without (A, C, E, G) or with 6 ng ml^−1^ RAD (B, D, F, H). After 72 h, the cells were subjected to fixing and SEM (2400×). Representative images were juxtaposed. The size bars represent 10 μm. I. Using the SEM images, the cell body lengths of 50 randomly selected cells were measured and compared. The Fig. 3 displays the total size range (bars), the 10–90 percentile (boxes) and the median (dividers in boxes). The synthesized Hsp90 variants are listed to the left. The significances of differences (p) are given to the right.

Exposure of *L. donovani* promastigotes to Hsp90 inhibitors GA and RAD induces a morphological conversion from promastigotes to amastigote-like cells that was ascribed to the inhibition of cytoplasmic Hsp90 (Wiesgigl and Clos, 2001). However, the possibility remained that GA might also target the endoplasmatic reticulum chaperone Grp96, a Hsp90 family member. We therefore tested the morphogenic impact of RAD on *L. donovani* bearing Hsp90 and Hsp90rr transgenes respectively. The result are displayed in Fig. 3. Before (Fig. 3A and C) or after (Fig. 3B and D) RAD treatment, *L. donovani* of either strain Ld[pTLv6:Hsp90] (Fig. 3A and B) or Ld[pTLv6:Hsp90rr] (Fig. 3C and D) were fixed and subjected to scanning electron microscopy. Both strains display typical promastigote shape in the absence of RAD (Fig. 3A and C). RAD exposure of Ld[pTLv6:Hsp90] cells caused the expected conversion towards an amastigote-like shape (Fig. 3B). No such morphogenic effects were observed with the Ld[pTLv6:Hsp90rr] parasites which maintained their promastigote-like shapes under RAD challenge (Fig. 3D).

We also measured the cell body length of ∼ 50 randomly selected cells from each culture (Fig. 3I). As expected, there is no difference between the two recombinant populations in the absence of RAD. Under RAD inhibition, the population expressing the wild-type Hsp90 transgene show a significant (*P* < 0.001) shortening of the cell body compared with the control culture without RAD. Expression of the Hsp90rr variant, in contrast, counteracts the effect of RAD on the cell body length (*P* < 0.0001) compared with the parasite line overexpressing the wild-type protein.

This result shows that the L33I exchange in Hsp90rr also abrogates the morphogenic effects of RAD on *L. donovani* promastigotes, excluding the ER chaperone Grp96 as target of RAD-induced stage conversion. Rather, the morphogenic impact is dependent on the susceptibility to RAD of the cytosolic Hsp90.

We have also established a system in which *L. donovani* depends on modified Hsp90rr transgenes for cell growth and maintenance of promastigote morphology. These transgenes can be modified further and transfected into *L. donovani* as episomes. The phenotype of any mutated form of Hsp90 will manifest itself only after RAD-mediated inhibition of the endogenously expressed Hsp90 pool.

### Impact of a putative Sti1 interaction motif

**Fig. 4:**
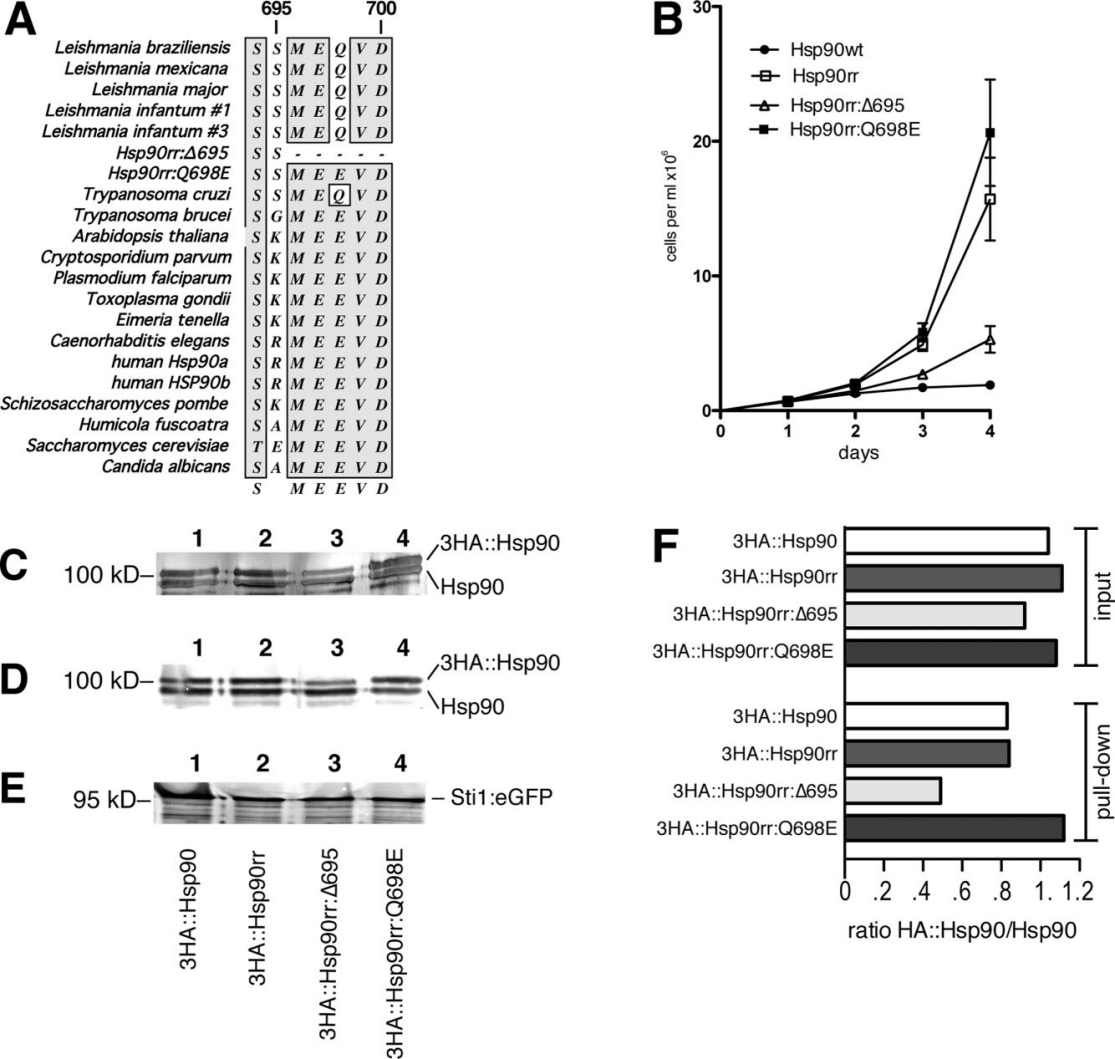
A. Alignment of the C′-terminal seven amino acids of *Leishmania* spp. Hsp90s with the orthologous sequences of Hsp90s from other species. The corresponding sequences of the Hsp90rr:Δ695 and Hsp90rr:Q698E mutants are also inserted. B. *In vitro* proliferation of promastigotes under 5 ng ml^−1^ RAD. Strains *L. donovani* [pTLv6:Hsp90] (solid circles), *L. donovani* [pTLv6:Hsp90rr] (open squares), *L. donovani* [pTLv6:Hsp90rr:Δ695] (open triangles) and *L. donovani* [pTLv6:Hsp90rr:Q698E] (solid squares) were seeded at 1 × 10^5^ ml^−1^ and grown at 25°C for 4 days. Cell density is plotted against time. C–E. Western blot analyses. *L. donovani* that overexpress the Sti1::eGFP fusion protein and one of the 3HA-tagged Hsp90 variants (3HA::Hsp90, lane 1; 3HA::Hsp90rr, lane 2; 3HA::Hsp90rr:Δ695, lane 3; or 3HA::Hsp90rr:Q698E, lane 4) were pre-treated for 72 h with 10 ng ml^−1^ RAD. C. Cell lysates analysed by SDS-PAGE and anti-Hsp90 Western blot. The upper bands represent the 3HA-tagged Hsp90 variants while the lower bands represent the endogenously coded Hsp90. D. GFP-Trap pull-down, followed by SDS-PAGE and anti-Hsp90 Western blot. The upper bands represent the 3HA-tagged Hsp90 variants while the lower bands represent the endogenously coded Hsp90. E. As (D), but probed with anti-Sti1 antibody. F. Quantification of the Western blots shown in (C) and (D). Ratio of the 3HA-tagged Hsp90 variants over the endogenously encoded Hsp90 are given for input (C) and GFT-trap-bound material (D).

Using this system, we proceeded to look into the interaction of Hsp90 with Sti1. *Leishmania* Hsp90 have an atypical Sti1 recognition motif (Fig. 4A). Instead of the C′-terminal MEEVD sequence common to the Hsp90 of the vast majority of organisms, all *Leishmania* Hsp90 carry the sequence MEQVD. We constructed expression plasmids (Fig. S3E) bearing Hsp90-coding sequences lacking this C′-terminal, presumed MEQVD motif (pTLv6:Hsp90rr:Δ695). We also created a variant of Hsp90rr with a Q698E exchange (pTLv6:Hsp90rr:Q698E), in effect creating the canonical MEEVD motif at the C′-terminus of the *L. donovani* Hsp90 (Fig. S3F). Expression of these Hsp90rr variants was also verified using qPCR (Fig. 2B). Differences were within the margin of error for qPCR.

Both variants, along with wild-type Hsp90 and Hsp90rr, were then tested for their effects on *L. donovani* growth under RAD. As shown in Fig. 4B, growth under RAD is significantly impaired with the Hsp90rr:Δ695 transgene that lacks the C′-terminal MEQVD motif. Interestingly, the Hsp90rr:Q698E carrying the ‘optimized’ MEEVD sequence shows even slightly better growth under RAD, arguing against a co-evolution of the MEQVD motif with its interacting co-chaperone(s) and their TPR domains.

Of course, our assumption that the MEQVD motif serves as Sti1 recognition motif is based on conclusion by analogy and requires experimental verification. Unfortunately, initial experiments showed that the Sti1–Hsp90 interaction is unaffected by RAD (data not shown). It was therefore impossible to disrupt Hsp90–Sti1 interaction and to selectively allow Hsp90rr–Sti1 binding. To circumvent this problem, we coexpressed the Hsp90rr variants with 27-amino-acid, N′-terminal triple HA tags (Fig. S4). These slightly elongated Hsp90rr variants were then coexpressed with a Sti1::eGFP fusion transgene (Fig. S5) that we had previously established to colocalize with Hsp90 (Fig. S5).

First, we established that the non-precipitated cell lysates contained comparable amounts of the various 3HA::Hsp90rr fusion proteins. We observe only negligible fluctuations between the different transgene-expressing parasite populations (Fig. 4C and F).

Next we tested the ability of the Sti1::eGFP fusion protein to interact stably with the 3HA::Hsp90rr variants. Using a GFPtrap kit, we precipitated Sti1::eGFP from the lysates of *L. donovani* promastigotes that coexpressed 3HA::Hsp90, 3HA::Hsp90rr, 3HA::Hsp90rr:Δ695 and 3HA::Hsp90rr:Q698E respectively. The pull-down material was analysed by SDS-PAGE and Western blot with anti-Hsp90 (D) and anti-Sti1 antisera (E). The latter shows a reproducible precipitation of Sti1::eGFP. As expected, both the endogenously encoded Hsp90 and the 3HA-tagged transgene products are co-precipitated with the Sti1::eGFP fusion proteins (D). Also as expected, the 3HA-tag Hsp90 variants form bands of slightly higher molecular mass that are distinct from the endogenously encoded Hsp90 (Fig. 4D). By quantifying relative band intensities and comparing the intensity ratios (Fig. 4F), we aimed to establish whether the C′-terminal Hsp90rr variants have an effect on the pull-down efficiency. Indeed, we find the pull-down of the 3HA::Hsp90rr:Δ695 deletion mutant impaired by almost 40% (Fig. 4D, lane 3; Fig. 4F). Conversely, the Q698E modification results in a slightly increased pull down ratio (Fig. 4D, lane 4; Fig. 4F), hinting at a slightly improved interaction with Sti1. Under the assumption that 3HA::Hsp90rr:Δ695 fusion proteins still form dimers with the endogenously coded Hsp90 and thus can be co-precipitated indirectly, the observed reduction of the pull-down ratio shows convincingly that the Δ695 deletion impairs Sti1 binding, establishing the MEQVD motif as Sti1 recognition signal.

Incidentally, the observed expression ratios for *Hsp90rr* versus endogenous *Hsp90* are in the range of 0.8–1.0, indicating that Hsp90 synthesis from the pTLv6-based episomes approaches natural Hsp90 synthesis.

The Hsp90–Sti1 interaction is also a key to the morphogenic development of *L. donovani*. Compared with *L. donovani* [pTLv6:Hsp90rr] which retains promastigote shape under RAD (Fig. 3D), the treatment of *L. donovani* [pTLv6:Hsp90rr:Δ695] results in the increased appearance of amastigote-like morphs (Fig. 3F). As expected, RAD has no morphogenic effects (Fig. 3H) on *L. donovani* [pTLv6:Hsp90rr:Q698E] parasites. This result indicates that the Sti1–Hsp90 interaction is crucial for the maintenance of the promastigote stage. Again, we confirmed the visual impression by performing cell body length measurements (Fig. 3I). Indeed, the lack of the C′-terminal MEQVD sequence causes a significant shortening of the cell bodies (*P* < 0.001), in keeping with a disruption of the crucial Hsp90rr–Sti1 binding.

### Hsp90–Sti1 binding is crucial for amastigote proliferation

Lastly, we tested the impact of the Hsp90–Sti1 interaction on the intracellular survival and proliferation of *L. donovani* in a bone marrow-derived macrophage (BMM) infection model. Since RAD will likely impact on mouse Hsp90 as well, it may interfere with macrophage functionality and thus compromise the results. Given previous experience (M. Wiesgigl, unpubl. results), we knew that *L. donovani* takes > 3 days to recover from RAD inhibition. We therefore treated the *L. donovani* promastigotes with RAD for 48 h prior to infection. The parasites were then added to the BMMs in fresh medium at a 10:1 ratio to prevent inadvertent exposure of the BMMs to RAD.

**Fig. 5:**
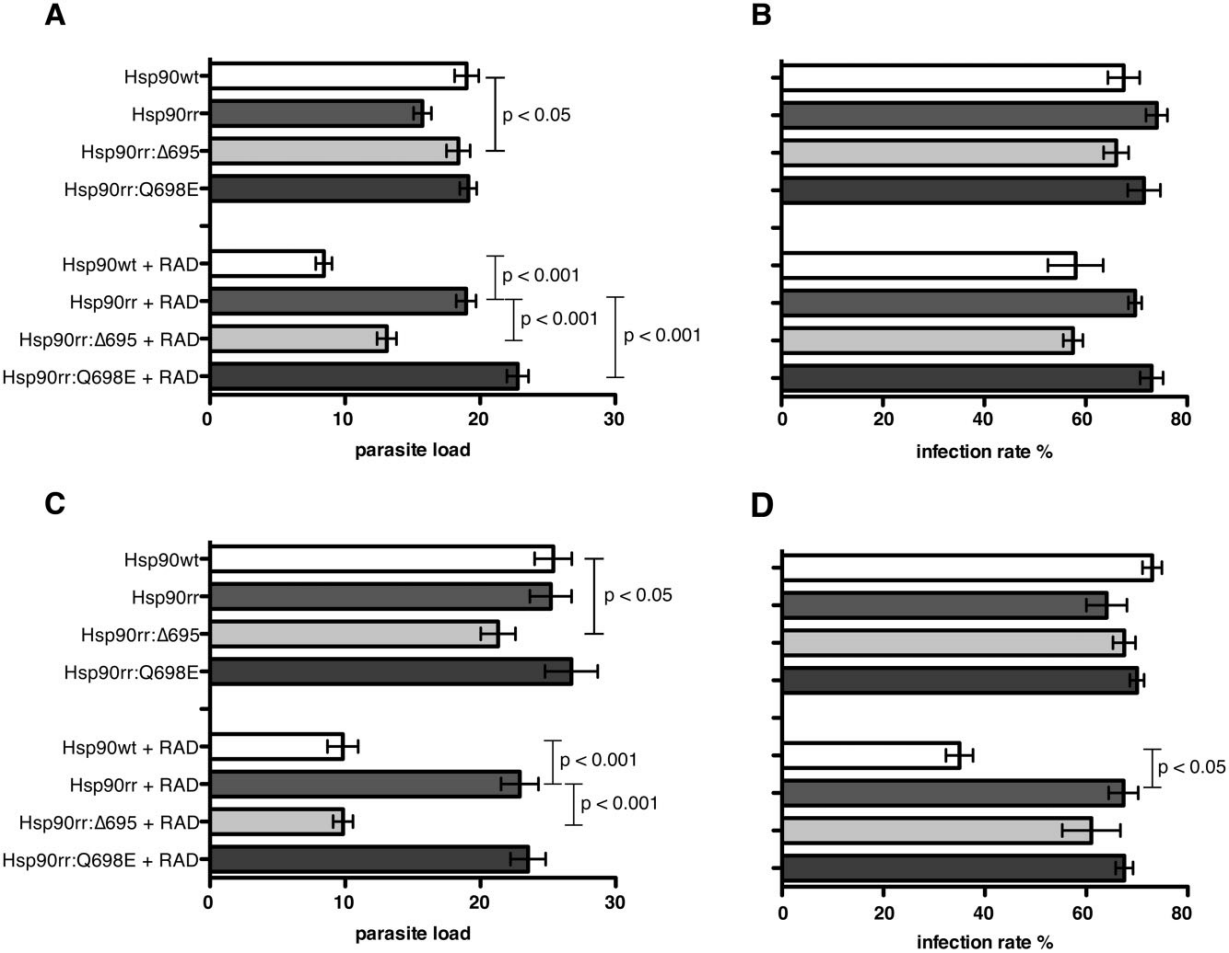
*In vitro* infection of bone marrow-derived macrophages with recombinant parasite populations expressing pTLv6:Hsp90, pTLv6:Hsp90rr, pTLv6:Hsp90rr:Δ695 and pTLv6:Hsp90rr:Q698E transgenes respectively. The parasites were either kept in RAD-free medium or pre-treated for 48 h with 6 ng (A, B) or 10 ng (C, D) ml^−1^ RAD (+RAD). Macrophages were then infected with the parasites at a 1:10 ratio. After 4 h, non-phagocytosed parasites were removed and the infected macrophage cultures were incubated at 37°C, 9% CO_2_, for another 20 (A, B) or 44 (C, D) h. Infected cells were fixed and stained with DAPI for fluorescence microscopy. Two hundred macrophages for each infection experiment were then counted for infection rate (per cent infected macrophages) and parasite load (# of parasites per macrophage). A Mann–Whitney *U*-test was applied to determine significance.

After 4 h, the unattached parasites were washed off, and the infected cells were incubated for another 20 and 44 h respectively. After DAPI staining, the infection rates (% infected macrophages) and the parasite loads (leishmaniae per macrophage) were determined by fluorescence microscopy. After 20 h, pre-treated Hsp90-overexpressing parasites caused mean parasite loads of seven leishmaniae per macrophage (Fig. 5A, Hsp90wt + RAD). In contrast, parasites with Hsp90rr (Fig. 5A, Hsp90rr + RAD) caused more than twice the intracellular parasite numbers at ∼ 17 per macrophage. Deletion of the MEQVD motif caused this to drop to ∼ 12 per macrophage (Fig. 5A, Hsp90rr:Δ695 + RAD), while the Q698E exchange resulted in ∼ 21 amastigotes per macrophage (Fig. 5A, Hsp90rr:Q698E + RAD).

These numbers show that while RAD pre-treatment of parasites does not abrogate infectivity completely, the observed drop is significant. The loss of the MEQVD motif and the concomitant weakening of Sti1 binding causes a reduction of the parasite load. Since infection rates were not significantly affected (Fig. 5B), the reduced parasite loads must be due to impaired amastigote proliferation. Once again, the parasites bearing the ‘optimized’ version of Hsp90 with the Q698E exchange outperformed those having the *Leishmania*-specific MEQVD pentapeptide. Continued incubation time (44h) did not affect the outcome in general. However, we observed a reduced infection rate for the parasites transfected with the Hsp90wt control (Fig. 5, Hsp90wt + RAD), indicating a loss of intracellular viability due to Hsp90 inactivation. Interestingly, loss of the MEQVD motif does not abrogate survival in macrophages since infection rates were unaffected (Fig. 5, Hsp90rr:Δ695 + RAD). However, the deletion of the MEQVD motif inhibited the intracellular proliferation of the parasites since parasite loads stagnated for this strain (Fig. 5C, Hsp90rr:Δ695 + RAD). Also, the initial advantage of the ‘optimized’ MEEVD motif is lost upon longer incubation (Fig. 5C, Hsp90rr + RAD versus Hsp90rr:Q698E + RAD).

We conclude that not only Hsp90 and Sti1 by themselves are crucial for *Leishmania*, but also their interaction. Given that Sti1 is the most conserved co-chaperone and usually required for the recognition of Hsp70/Hsp40/client complexes, this finding shows that Sti1 functionality was established early in the development of eukaryotes before the euglenozoa branched off the crown group stem.

## Discussion

### Sti1 is essential for promastigote viability

Two lines of evidence now indicate an essential role for the co-chaperone Sti1. First, it was shown that a counter-selectable, episomal transgene of Sti1 in a Sti1 gene replacement (Sti1^−/−^) background could only be eliminated in the presence of other, functional transgene copies (Morales *et al*., 2010). Moreover, the mutation of a single phosphorylation site rendered the additional transgene incapable of supporting viability. Second, this analysis shows that a double allelic gene replacement is feasible only in the presence of a functional Sti1 transgene. Attempts to replace both natural alleles in the absence of an additional gene copy resulted in dual-resistant promastigotes which had nevertheless retained gene copies, presumably the result of spontaneous, episomal gene amplification events.

Moreover, we observe a pronounced colocalization of Sti1 with Hsp90 and Hsp70, in keeping with these proteins' participation in foldosome function. It may also be concluded from our results that the gene annotated as second Sti1/HOP orthologue, HOP2, is functionally distinct from the Sti1 gene as its presence is insufficient to overcome a lack of Sti1. This all the more since the HOP2 gene can be subjected to double-allelic gene replacement without phenotypic consequence *in vitro* (Ommen *et al*., 2009).

### Hsp90 is a difficult target for reverse genetics

Sti1 is believed to exert its function through its interaction with major chaperones such as Hsp90 and Hsp70. While an association of Sti1 with both chaperones has been proven in *Leishmania* spp. (Webb *et al*., 1997; Ommen *et al*., 2010), the impact of such an interaction has not been clear. The use of a counter-selectable Sti1 expression vector in Sti1^−/−^ mutants can clear up the structural requirements from the Sti1 side. However, such an approach is not feasible for Hsp90 or Hsp70 since both proteins are encoded by multiple, tandemly arranged, identical gene copies in *Leishmania* spp. (Lee *et al*., 1988; Hubel and Clos, 1996; Shapira *et al*., 2001). So far, no tandem arrays of genes have been successfully targeted for replacement in *Leishmania* spp.

The *C. elegans* Hsp90 was described as resistant to the inhibitor GA (David *et al*., 2003), opening the possibility to express a GA-resistant Hsp90 while blocking the endogenously encoded Hsp90 with the drug. However, the structural basis for the GA resistance of *C. elegans* Hsp90 is not known. Moreover, the *C. elegans* protein does not render *S. cerevisiae* resistant to the drug indicating at least phylum specificity of Hsp90 function.

### A CKC strategy for *L. donovani* Hsp90

The recent discovery of a RAD-resistant Hsp90 in the fungus *H. fuscoatra* and the identification of a single amino acid exchange as basis for this resistance (Prodromou *et al*., 2009) paved the way for our analysis of Hsp90/Sti1 interaction. The complementation/chemical knockout (CKC) regimen indeed worked out as planned. Surprisingly, the exchange of a single leucine against its isomer isoleucine at position 33 in the *L. donovani* Hsp90 results in a functional, RAD-resistant form of the protein (Hsp90rr) that can support culture growth under normally inhibiting IC_90_ for the drug. This is not due to a sequestration effect. First, the equivalent overexpression of the natural Hsp90 does not interfere with RAD efficacy. Second, we can also exclude that Hsp90rr sequesters the drug and thus alleviates inhibition of the endogenously coded Hsp90 – if this were the case, then the C′-terminal deletion mutant LdHsp90rr:Δ695 would also support growth under RAD.

We have therefore created a system in which we can test the phenotypic effects of Hsp90 mutations. The phenotypes only come to bear under RAD-mediated inhibition of the pool of natural Hsp90. The phenotype of two mutations affecting Sti1 interaction with Hsp90, namely deletion of the C′-terminal MEQVD motif or the change to an MEEVD motif, was observed only under RAD challenge. This opens up possibilities to study other functional motifs and domains of *Leishmania* Hsp90, namely phosphorylation sites (Morales *et al*., 2010) and amino acid residues with impact on ATPase activity.

### The C′-terminal MEQVD motif is critical for Hsp90 function

The C′-terminal MEQVD motif may interact with TPR-containing co-chaperones other than Sti1. Hsp90 complexes contain a dimer of Hsp90, and recent results suggest that only one Hsp90 subunit is bound by Sti1 (Li *et al*., 2011). Another candidate may be the *L. donovani* SGT (small glutamine-rich TPR protein). We could recently show LdSGT as being essential for growth and associated with Sti1, Hsp90 and Hsp70. However, LdSGT is dimeric within the Hsp90-containing complexes of *Leishmania* (Ommen *et al*., 2010) and generally thought to interact with Hsp90 through Hsc70. The other TPR-containing co-chaperones examined so far are not essential for growth, e.g. HIP and HOP2 (Ommen *et al*., 2009) or cyclophilin 40 (W.-L. Yau *et al*., unpubl. work). Thus, the probability that the Hsp90/Sti1 interaction causes the phenotype of the Hsp90rr:Δ695 mutant is high.

### A variant Sti1 recognition motif in *Leishmania* spp

We also made the curious observation that the MEQVD motif at the C′-terminus of all *Leishmania* Hsp90s is a suboptimal variant of the conventional MEEVD motif. Given that Sti1 is known as inhibitor of Hsp90 ATPase activity (Prodromou *et al*., 1999; Li *et al*., 2011), the less effective binding may result in an elevated ATPase activity by *Leishmania* Hsp90. Interestingly, the MEEVD sequence motif provides *L. donovani* with a slight advantage in the early stage of the infection (Fig. 5A and B). So far, we have no plausible explanation for this finding. *Trypanosoma cruzi*, another intracellular parasite of the Trypanosomatidae family, carries the same modified C′-terminal Sti1 recognition motif (MEQVD) while *Trypanosoma brucei*, causative agent of African sleeping sickness and proliferating extracellularly in the bloodstream, has the canonical MEEVD motif. In this context it may be interesting to test the impact of a Hsp90 with an MEEVD C′-terminus on *T. cruzi* virulence.

However, other intracellular pathogens such as *Plasmodium falciparum* possess the standard MEEVD sequence (Fig. 4A). The difference may be that *Leishmania* and *T. cruzi* infections are chronic in nature, much in contrast to malaria which is an acute infection. *Leishmania* spp. depend on long periods of survival within mammalian hosts for their infection cycle since the transmitting sandflies have no year-round activity. It is possible that too much intracellular proliferation of *Leishmania* spp. may overwhelm the host's immune defence prematurely, cause exacerbated disease forms and progression, and thus affect the chain of infection negatively. The same would be true for *T. cruzi* where, after an acute phase, the parasites must persist within the host for decades. An exacerbation of the acute phase might also affect the overall parasite survival negatively.

### Take-home message and outlook

This first use of a CKC strategy yielded several functional results: (i) the Sti1 interaction motif in Hsp90 is crucial for growth of the promastigote and the amastigote stage, (ii) it is indeed the inhibition of cytosolic Hsp90 that mimics the environmental stimuli for conversion to an amastigote-like morphology, and (iii) inhibition of Hsp90 by RAD is not stochiometric since the overexpression of the drug-sensitive variant does not change the sensitivity for the drug. The latter result differs from the observations made for the Hsp90 inhibitor GA. Here, the drug effect is counteracted by Hsp90 overexpression, both via episomal transgenes (Fig. 2D) (Wiesgigl and Clos, 2001), and via spontaneous gene amplification (Wiesgigl and Clos, 2001).

We are confident that the CKC system will allow a detailed analysis of functional Hsp90 motifs and of post-translational modification events (Morales *et al*., 2008; 2010). It becomes clear that the Hsp90 foldosome complex is an integral part of the signal transduction pathways in *Leishmania* spp. It will be of great interest to test the impact of phosphosite mutations on Hsp90 function, but also to see the impact of Hsp90 mutations on protein kinase activities in these phylogenetically ancient eukaryotic microorganisms. Furthermore, it will be of great interest to dissect basic chaperone activities and complex foldosome functionality in the context of Hsp90-mediated drug resistance (Vergnes *et al*., 2007).

Moreover, it is tempting to envision a replacement of the Hsp90 multi gene cluster by homologous recombination and in the presence of the Hsp90rr transgene. The often observed spontaneous amplification of targeted genes would not produce a selective advantage to the recombinant parasites under radicicol selection pressure. This would increase the likelihood of obtaining complete replacement of the endogenous Hsp90 gene copies and preclude the formation of mixed Hsp90 complexes between endogenous wild-type protein and the episomally encoded Hsp90rr variants. It would also give an answer to the question whether multi-gene clusters can be targeted for homologous recombination in *Leishmania*.

## Experimental procedures

### Sequence analysis

All sequence comparisons and *in silico* plasmid construction was performed using the MacVector software suite, versions 11.0 to 12.5. The TriTrypDB database was used as reference for *Leishmania* gene sequences.

*Arabidopsis thaliana* NP_200076.1; *Caenorhabditis elegans* NP_506626.1; *Candida albicans* XP_721353; *Cryptosporidium parvum* XP_626924.1; *Eimeria tenella* AF042329; *human Hsp90a* NM_001017963; *human Hsp90b* NM_003299; *Humicola fuscoatra* EU747829; *Leishmania braziliensis* LbrM.33.0350; *Leishmania infantum #1* LinJ.33.0350; *Leishmania infantum #3* LinJ.33.0360; *Leishmania mexicana* LmxM.32.0312; *Leishmania major* LmjF.33.0312; *Plasmodium falciparum* PF3D7_0708400; *Saccharomyces cerevisiae* AAA02743.1; *Schizosaccharomyces pombe* NP_594365.1; *Toxoplasma gondii* AY292379; *Trypanosoma cruzi* Tc00.1047053509105.140; *Trypanosoma brucei* Tb427.10.10890.

### *L. donovani* strain and cultivation

Promastigote stages of *L. donovani* 1SR were grown at 25°C in supplemented M199 medium (Hubel *et al*., 1997). After electroporation, recombinant parasites were cultivated in supplemented Medium199 with the appropriate selection antibiotics. For routine cultivation, cells were grown to late log phase in order to maintain exponential growth. Cell density was monitored using a Schaerfe System CASY cell counter.

Hsp90 inhibitors RAD and GA were purchased from Sigma-Aldrich (München, Germany) and CAYLA-Invivogen (Toulouse, France) respectively. New batches of inhibitors were first tested to establish the batch-specific IC_90_.

### Western blot analysis

Antibodies against Hsp90 were produced as polyclonal sera in mice (Ommen *et al*., 2010). Production of SDS cell lysates, discontinuous SDS-PAGE and Western blot were performed according to standard protocols. Briefly, membranes were treated with blocking solution (5% milk powder and 0.1% Tween 20 in Tris-buffered saline), before they were probed using the polyclonal antibody fraction (1:1000 in blocking solution), followed by incubation with an anti-mouse IgG-alkaline phosphatase conjugate (1:1000 in blocking solution) as secondary antibody. Blots were developed using nitro blue tetrazolium chloride and 5-bromo-4-chloro-3-indolyl phosphate.

Animal care and experimentation were performed in accordance with the German Federal Animal Protection Laws, in particular §§4, 7 and 10a, in the Animal Facility of the Bernhard Nocht Institute.

### Targeted mutagenesis of Hsp90-coding sequences

To create point mutations and deletion mutations, we used a modified PCR-based strategy. First, the Hsp90-coding sequence was amplified from *L. donovani* 1SR gDNA using primers Hsp90.Kpn.fwd (GGGGTACCATG ACGGAGACGT TCGCGTTC) and Hsp90.Bam.rev (GGGGGATCCT CAGTCCACCT GCTCCATG) creating KpnI and BamHI sites at the 5′ and 3′ ends respectively. The PCR product was then cut with KpnI and BamHI and ligated into the plasmid pUC19 also linearized with KpnI and BamHI. Plasmid pUC:Hsp90 then served as template for site-directed mutagenesis (Fig. S6). Primers Hsp90.L33I.fwd (CGCGAGATCA TCAGCAATGC GTCGGATG) and Hsp90.L33I.rev (CAGGAAGATC TCCTTGTTCG) were phosphorylated using ATP and polynucleotide kinase (PNK) and then used to prime a PCR amplification of pUC:Hsp90 using the iProof-PCR kit for GC-rich DNA (#172-5320) from Bio-Rad Laboratories (München, Germany). Following amplification (30 cycles), the linear PCR product was subjected to recircularization using ligase (3 h, RT). This pUC:Hsp90rr was used to transform competent *Escherichia coli* DH5-α cells (#18265-017, Invitrogen, Karlsruhe, Germany). After amplification and purification (Sambrook and Russell, 2001) via caesium-chloride density gradient ultracentrifugation (50% w/v CsCl, 6h, 90 000 r.p.m., 20°C, rotor NVT90, Beckman, Krefeld, Germany), the pUC:Hsp90rr was verified by DNA sequencing (LGC Genomics, Berlin, Germany).

Plasmid pUC:Hsp90rr was used as template for derived mutants. Using primers Hsp90.Kpn.fwd (GGGGTACCATG ACGGAGACGT TCGCGTTC) and Hsp90.Δ695.Bam.rev (GGGGGATCCT AGCTGGAGGT GCCGGCGGTG), a Hsp90rrΔ695-coding sequence was amplified and subcloned into pUC19, creating pUC:Hsp90rrΔ695. To create a Q698E variant of Hsp90rr, pUC:Hsp90rr was amplified using the phosphorylated primers Hsp90.Q698E.fwd (AGCATGGAGG AGGTGGACTG AGGATCCTCTAG) and Hsp90.Q698E.rev (GGAGGTGCCG GCGGTGACC). The linear PCR product was circularized with ligase (pUC:Hsp90rr:Q698E) and used to transform *E. coli*.

### Vector pTLv6

The 3507 bp SacI–SalI fragment from pcosTL (Kelly *et al*., 1994) was cloned between the SacI and SalI sites of pUC19 (Yanisch-Perron *et al*., 1985) to create pTLv2. After excising a 300 bp HindIII fragment of mostly *T. cruzi* GA3PDH 3′-UTR, the ends were filled with Klenow enzyme and religated creating the pTLv3 plasmid that lacks HindIII sites. Plasmid pTLv3 was then opened with BamHI to remove the pUC19 multiple cloning site and most of the *T. cruzi* Ga3PDH 5′-UTR sequences. Then the 2246 bp BamHI fragment from the integration vector pIRmcs3+ (Hoyer *et al*., 2004) was ligated in to create pTLv6 (Fig. S3A) which was then linearized using enzymes KpnI and BglII.

### Plasmid pTLv6:Hsp90

Hsp90-coding sequences from pUC:Hsp90, pUC:Hsp90rr, pUC:Hsp90rr:Δ695, and pUC:Hsp90rr:Q698E were excised with enzymes KpnI and BamHI (compatible with BglII sticky ends) and ligated into the linearized pTLv6 vector to create pTLv6:Hsp90, pTLv6:Hsp90rr, pTLv6:Hsp90rr:Δ695, and pTLv6:Hsp90rr:Q698E (Fig. S3C–F).

### Plasmid pCLneo:3HA::Hsp90

To create pCLneo (D. Zander, unpubl. data) the second NdeI restriction site in the vector backbone of pTLv6 was removed by targeted mutagenesis (see above). For this, the primers pTLv8.fwd (GTGGGTGGAC GCAGCGATG) and pTLv8.rev (ACGAAAGGGC CTCGTGATAC) were used to create a linear PCR product that was circularized to create pCLneo.

The KpnI–BamHI fragment from pIR:3HA (M. Chrobak, unpubl. data) was inserted into pCLneo to create pCLneo:3HA (Fig. S4). The various Hsp90-coding sequences were then amplified using primers Hsp90.NdeI.fwd (GGGCATATGA CGGAGACGTT CGC) and Hsp90.Bam.rev (GGGGGATCCT CAGTCCACCT GCTCCATG), cut with NdeI and BamHI and ligated into pCLneo:3HA to create pCLneo:3HA:Hsp90, pCLneo:3HA:Hsp90rr, pCLneo:3HA:Hsp90rr:Δ695 and pCLneo:3HA:Hsp90rr:Q698E (Fig. S4B–E). All relevant gene sequences were verified by DNA sequencing.

### Plasmid pCLsat:Sti1::eGFP

The neomycin phosphotransferase-coding sequence of pCLneo was eliminated by targeted mutagenesis and replaced with the strepthricine acetylase-coding sequence yielding pCLsat (D. Zander, unpubl. data). To create pCLsat:Sti1::eGFP (Fig. S5), pCLsat and pTLv4:Sti1::eGFP (G. Ommen, unpubl. data) were cut with KpnI and BamHI. The fragment containing the Sti1::eGFP-coding sequence was ligated into the linearized pCLsat vector, and the proper insertion was verified by DNA sequencing.

### Electrotransfection and selection

Electrotransfection of *Leishmania* promastigotes was carried out essentially as described (Krobitsch *et al*., 1998). *Leishmania* parasites were taken from late log phase cultures (1–2 × 10^7^ ml^−1^), sedimented (720 *g*, 8 min, 4°C), washed twice with ice-cold PBS and once in pre-chilled electroporation buffer (Laban and Wirth, 1989). Cells were resuspended in electroporation buffer at a density of 1 × 10^8^ ml^−1^. Aliquots of 0.4 ml were mixed with 50 μg of circular DNA for stable episomal transfection. Electroporation was carried out in pre-chilled 4 mm electroporation cuvettes in a Bio-Rad Gene Pulser, with three pulses at 1.5 kV (3750 V cm^−1^), 25 μF and 200 ohm. A mock transfection was performed in identical fashion, but without DNA, to provide negative control strains for the antibiotic selection.

Following electroporation, cells were kept on ice for 10 min before they were transferred to 10 ml of drug-free medium. Bleomycin (5 μg ml^−1)^, puromycin (25 μg ml^−1^), G418 (50 μg ml^−1^) or nourseothricine (150 μg ml^−1^) was added after 24 h, and cultivation was continued until the mock transfection population had succumbed to the antibiotic pressure. For cloning, promastigotes were seeded in supplemented Medium199 at 0.5 cells per well in microtitre plates. After 10–14 days, wells positive for promastigote growth were identified and their content transferred to culture flasks.

### Expression profiling

Semi-quantitative real-time RT-PCR was performed essentially as described (Choudhury *et al*., 2008). Hsp90 transgene-specific primers were pTLv6.Hsp90.F2 (CGACGAGGAG GAGGAGGCAG) and pTLv6.Hsp90.B1 (GCCAGTACAT CACAAGACTC ATAGATCC). Hsp90 mRNA abundance was calculated relative to the actin signal.

### *In vitro* infection experiments

Murine bone marrow macrophages (BMM) were isolated from the femurs of female C57BL/6 mice and differentiated in Iscove's modified Dulbecco's medium (IMDM) supplemented with 10% heat-inactivated FCS, 5% horse serum and 30% L929 supernatant (modified after Racoosin and Beverley, 1997). For infection, BMM were harvested, washed and seeded into the wells of an eight-well chamber slide (NUNC) at a density of 2 × 10^5^ cells per well. The macrophages were incubated for 48 h at 37°C and 9% CO_2_ to allow adhesion of the cells. The promastigotes expressing the different Hsp90 variants (pTLv6:Hsp90, pTLv6:Hsp90rr, pTLv6:Hsp90rr:Δ695, pTLv6:Hsp90:Q698E) were exposed to 6 ng ml^−1^ RAD prior to infection for 48 h. Adherent BMM were infected with stationary-phase promastigotes (Racoosin and Beverley, 1997) at a multiplicity of infection of 10 parasites per macrophage. After 4 h of incubation at 37°C in modified Medium199, non-phagocytosed parasites were removed by multiple washing steps with PBS and incubation was continued for another 20 or 44 h in IMDM at 37°C and 9% CO_2_. The medium was removed. The cells were washed twice and fixed in ice-cold methanol. Intracellular parasites were quantified by nuclear staining with DAPI (1.25 μg ml^−1^, Sigma) and fluorescence microscopy.

### Immunofluorescence and confocal microscopy

Log-phase promastigotes (1 × 10^7^ cells) were sedimented by gentle centrifugation (750 *g*), washed twice with phosphate-buffered saline (PBS) and suspended in 1 ml of PBS. Aliquots (2 × 10^5^ cells) of the suspension were applied on poly-l-lysine-coated microscopic slides. After fixing the cells for 2 min in ice-cold methanol, the slides were air-dried for 20 min. Non-adherent cells were removed by a gentle wash (0.1% Triton X-100 in PBS) followed by incubation in blocking solution (2% BSA, 0.1% Triton X-100 in PBS). Slides were then incubated for 1 h with mouse anti-Sti1 diluted 1:250 in blocking solution and with either chicken anti-Hsp70 or chicken anti-Hsp90 (Hubel *et al*., 1995). Cells were washed thrice and then incubated for 1 h with anti-mouse (goat) secondary antibody coupled to FITC (Dianova, 1:250), with anti-chicken-Alexa594 (Dianova, 1:500) and with DAPI (1:25). After washing the slides thrice, Mowiol and coverslips were applied and the slides were left to dry for 24 h at 4°C.

Fluorescence microscopy was carried out on an Olympus FluoView1000 confocal microscope (SIM-scanner and spectral detection).

### Immune precipitation

Promastigote cells of *L. donovani* [pCLsat:Sti1::eGFP] coexpressing Hsp90rr variants tagged with three tandem, N′-terminal haemagglutinin (HA) epitopes were exposed to 10 ng ml^−1^ RAD for 72 h. Promastigote cells (1 × 10^7^) were washed twice in pre-chilled PBS and homogenized in 100 μl of cell lysis buffer (10 mM Tris/HCl, pH 7.5, 150 mM NaCl, 0.5 mM EDTA, 0.5% (v/v) Triton X-100, 1 mM PMSF, 0.5 mM 1,10-Phenanthrolin). After incubation on ice for 30 min, lysates were centrifuged (20 000 *g*, 4°C, 10 min), and the supernatant was adjusted to a volume of 400 μl with dilution buffer (10 mM Tris/HCl, pH 7.5, 150 mM NaCl, 0.5 mM EDTA, 1 mM PMSF, 0.5 mM 1,10-Phenanthrolin). Aliquots of this fraction (50 μl) were mixed at 1:1 ratio (v/v) with 2× SDS sample buffer (‘input’ fraction). For pull-down of immune complexes, 25 μl of GFP-TrapM® Beads (Chromotek, Germany) were washed twice with ice-cold dilution buffer, mixed with cell lysate and incubated for 2 h at 4°C under agitation. After magnetic precipitation using a magnetic particle concentrator (Dynal), 50 μl of supernatant was mixed with 2× SDS sample buffer (non-bound fraction). The GFP-Trap beads were washed twice in dilution buffer, suspended in 1× SDS sample buffer and heated to 95°C for 10 min. Beads were sedimented magnetically (4°C, 2 min), and SDS-PAGE was performed with the supernatant (‘bound’ fraction) along with the ‘input’ and ‘non-bound’ fractions. Separated proteins were then stained by immune blot analysis using anti-Hsp90 serum. After developing the immune blots with NBT and X-phosphate (Hubel *et al*., 1995), the band intensities were quantified using the ImageJ software.

### Scanning electron microscopy

*Leishmania* cells were washed twice in PBS, fixed in 2% glutaraldehyde in sodium cacodylate buffer and post-fixed with 1% osmium. Samples were dehydrated at increasing ethanol concentrations (30–100%). After critical point drying, samples were treated with gold and analysed on a Philips SEM 500 electron microscope. Images were taken using a conventional 35 mm camera, and the developed black-and-white films were digitalized using a HAMA 35 mm film scanner. After importing the digital images into the Intaglio graphics software (Purgatory Design) we performed the length measurements using the measurement line tool. Measurements in centimetres were then normalized using the integrated size bars.

### Statistical analyses

Data were collected using the Prism software package (Graph Pad, version 5). Significance values were determined using the Mann–Whitney *U*-test (Mann and Whitney, 1947).

## Supporting information

**Fig. S1.** A. Schematic representation of the *Sti1* replacement constructs. Approximately 1000 bp of *Sti1* 5′-UTR were amplified by PCR with EcoRI, SwaI and KpnI sites added as indicated and ligated between EcoRI and KpnI sites of plasmid pUC19 to produce pUC19- Sti1-5′. Similarly, the 3′-UTR of *Sti1* was amplified with BamHI, SwaI and HindIII sites and integrated between the BamHI and HindIII sites of pUC19-Sti1-5′ to create pUC19- Sti1-5′-3′. Next, the selection marker genes for puromycin (puro) and bleomycin (bleo) were fitted between the KpnI and BamHI sites to produce the two replacement constructs pUC19-Sti1-5′-puro-3′ and pUC19-Sti1-5′bleo-3′ respectively. Prior to electroporation, the gene replacement cassettes of either construct were excised using SwaI restriction digest.

B. Schematic representation of the gene add-back construct pIRmcs3+ Sti1. The open reading frame plus 1000 bp of the 3′-UTR from the Sti1 gene were amplified to create KpnI and BglII sites at either ends and ligated between the KpnI and BglII sites of pIRmcs3+. Digesting the plasmid with SwaI yielded a linearized fragment with small subunit rRNA sequences on either ends. After electroporation, the gene expression cassette is integrated into one of the SSU rRNA-coding sequences.

**Fig. S2.** PCR analysis of putative *Sti1* replacement mutants.

A. Amplification of open reading frame sequences by PCR. Genomic DNA from four suspected Sti1^−/−^ gene replacement mutants was used as template. Note that all four clones still possess Sti1-coding sequences.

B. Amplification of the *Sti1* locus including the flanking DNA sequences. Genomic DNA from *L. donovani* 1SR wild-type (lane 1), *L. donovani* Sti1^+/−^ (lane 2) and of six suspected Sti1^−/−/+^ gene replacement/gene add-back clones (lanes 3–8) was used as template. Note that the ∼ 3600 bp species representing the wild-type locus is lacking from lanes 3–8, indicating that replacement of the *Sti1-*coding sequences was successful in the presence of a *Sti1* transgene. As expected, the mono-allelic gene replacement mutant Sti^+/−^ (lane 2) shows a mixed band pattern representing one wild-type and one recombined *Sti1* locus.

C. Schematic annealing of the PCR primers used.

Sti1_locus_fwd (GGTGACCGCGAGCGGCTTC)

Sti1_ORF-5′ (CCATGGTACCATGGACGCAACTGAGCTG)

Sti1_ORF_3′ (CCTCTAGATCTCTACTGACCAAAACGAATG)

Sti1_locus_rev (CTGCCAACGTGACACCTG).

**Fig. S3.** Schematic representation of *Hsp90* transgenes. The plasmid vector pTLv6 (A) was combined from the cosmid pcosTL (Kelly *et al*., 1994) and pIRmcs3+ (Hoyer *et al*., 2004) and allows the stable episomal establishment of transgenes in *Leishmania* spp. Coding sequences for Hsp90 variants were excised from pUC19 with KpnI and BamHI and fused between the KpnI and BglII sites of pTLv6 (B). The Hsp90 variants are schematically drawn up with mutation sites highlighted (C–F).

**Fig. S4.** Schematic representation of 3xHA-tagged Hsp90 transgenes. The plasmid vector pCLneo:3HA (A) was opened with NdeI and BglII. The coding sequences for the Hsp90 variants were amplified by PCR to create NdeI and BamHI sites, subcloned into pUC19 and verified, and then fused between the NdeI and BglII sites of pCLneo:3HA. The Hsp90 variants are schematically drawn up with mutation sites highlighted (B–E).

**Fig. S5.** Upper panel: Schematic representation of pCLsat:Sti1::eGFP. The streptothricine (SAT) resistance marker expression is driven by *T. cruzi* glyceraldehyde 3′ phosphate dehydrogenase gene flanking sequences. Sti1::eGFP fusion gene expression is under control of the *L. mexicana* cystein proteinase B2 intergenic region and the *L. donovani LPG1* 3′ UTR. Lower panel: Fluorescence microscopy imaging of Sti1::eGFP and anti-Hsp90 immune staining.

**Fig. S6.** Schematic representation of primer directed mutagenesis. Hsp90-coding sequences were amplified and ligated into plasmid pUC19. A forward primer bearing the desired mutation six base pairs from its 5′ end anneals immediately downstream from a reverse primer with wild-type sequence (A). Both primers were 5′-phosphorylated prior to PCR using polynucleotide kinase. Amplification using the iProof kit with the buffer for G/C-rich DNA yielded a linear product (B) bearing the planned mutation. Ligation of the blunt ends then produced the pUC-Hsp90 plasmid with the mutation (C). Using KpnI and BamHI, the coding sequence was excised for insertion into the expression vector pTLv6 (SM3).
