# Supplementary material for: The Hsp90–Sti1 interaction is critical for Leishmania donovani proliferation in both life cycle stages
Source: Cell Microbiol. 2012 Nov 20;15(4):585–600. doi: 10.1111/cmi.12057 (PMC3654555; doi:10.1111/cmi.12057)
Supplement: Supplementary file 1 [file cmi0015-0585-SD1.pdf]

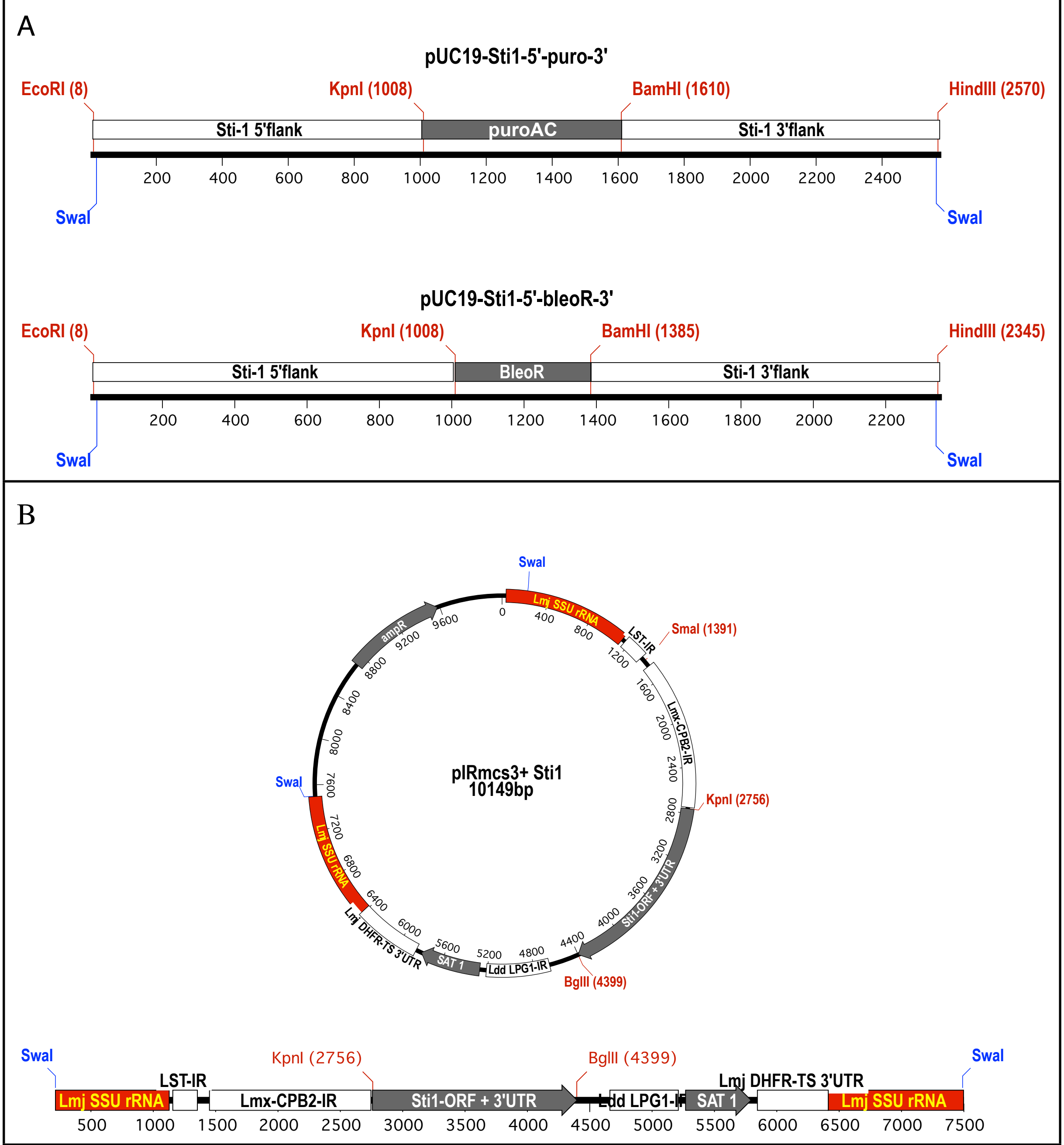

SM1: (A) Schematic representation of the *Sti1* replacement constructs. Approximately 1000 bp of *Sti1* 5'-UTR were amplified by PCR with *Eco*RI, *Swa*I and *Kpn*I sites added as indicated and ligated between *Eco*RI and *Kpn*I sites of plasmid pUC19 to produce pUC19-Sti1-5'. Similarly, the 3'-UTR of *Sti1* was amplified with *Bam*HI, *Swa*I and *Hin*dIII sites and integrated between the *Bam*HI and *Hin*dIII sites of pUC19-Sti1-5' to create pUC19-Sti1-5'-3'. Next, the selection marker genes for puromycin (puro*)* and bleomycin (bleo) were fitted between the *Kpn*I and *Bam*HI sites to produce the two replacement constructs pUC19-Sti1-5'-puro-3' and pUC19-Sti1-5'bleo-3', respectively. Prior to electroporation, the gene replacement cassettes of either construct were excised using *Swa*I restriction digest. (B) Schematic representation of the gene add-back construct pIRmcs3+ Sti1. The open reading frame plus 1000 bp of the 3'-UTR from the Sti1 gene were amplified to create *Kpn*I and *Bgl*II sites at either ends and ligated between the *Kpn*I and *Bgl*II sites of pIRmcs3+. Digesting the plasmid with *Swa*I yielded a linearised fragment with small subunit rRNA sequences on either ends. After electroporation, the gene expression cassette is integrated into one of the SSU rRNA coding seuquences.

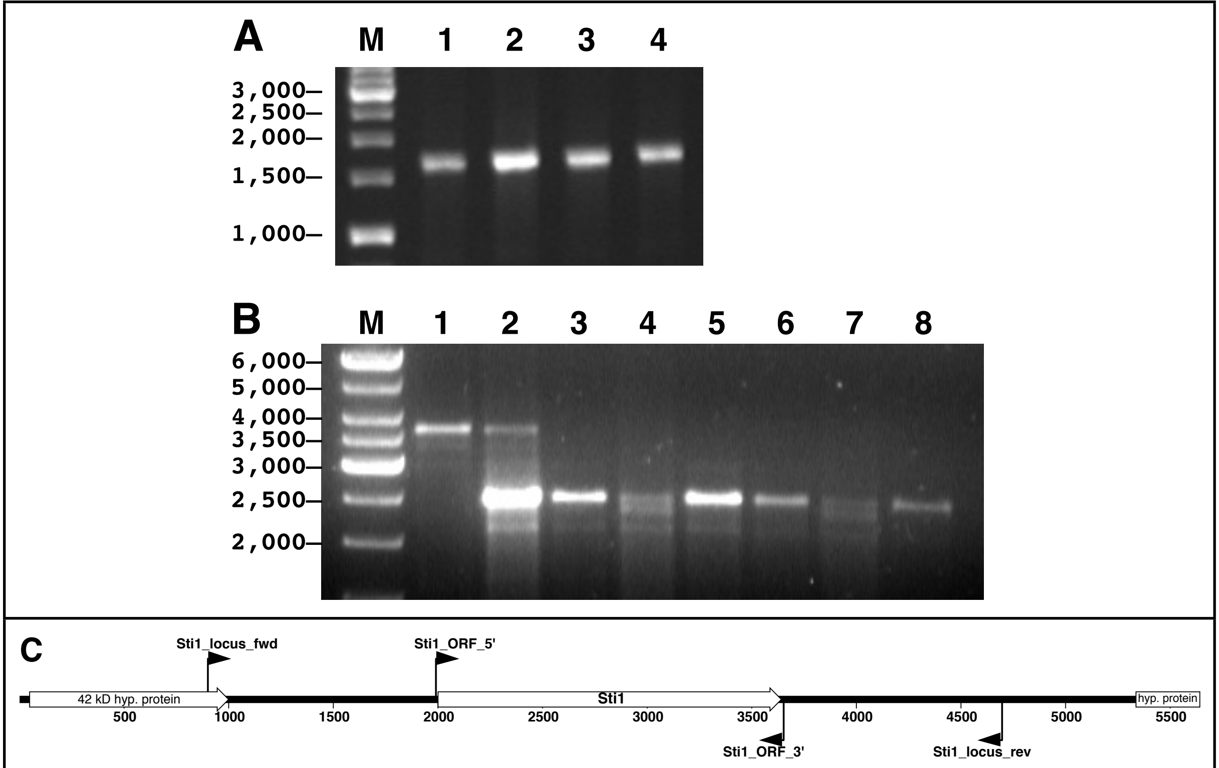

PCR analysis of putative *Sti1* replacement mutants. (A) Amplification of open reading frame sequences by PCR. Genomic DNA from 4 suspected Sti1$^{-/-}$ gene replacement mutants was used as template. Note that all 4 clones still possess Sti1 coding sequences. (B) Amplification of the *Sti1* locus including the flanking DNA sequences. Genomic DNA from *L. donovani* 1SR wild type (lane 1), *L. donovani* Sti1+/- (lane 2) and of 6 suspected Sti1$^{-/-/+}$ gene replacement/gene add-back clones (lanes 3-8) was used as template. Note that the ~3,600 bp species representing the wild type locus is lacking from lanes 3-8, indicating that replacement of the *Sti1* coding sequences was successful in the presence of a *Sti1* transgene. As expected, the mono-allelic gene replacement mutant Sti$^{+/-}$ (lane 2) shows a mixed band pattern representing one wild type and one recombined *Sti1* locus. (C) Schematic annealing of the PCR primers used.

Sti1_locus_fwd (GGTGACCGCGAGCGGCTTC)
Sti1_ORF-5' (CCATGGTACCATGGACGCAACTGAGCTG)
Sti1_ORF_3' (CCTCTAGATCTCTACTGACCAAAACGAATG)
Sti1_locus_rev (CTGCCAACGTGACACCTG).

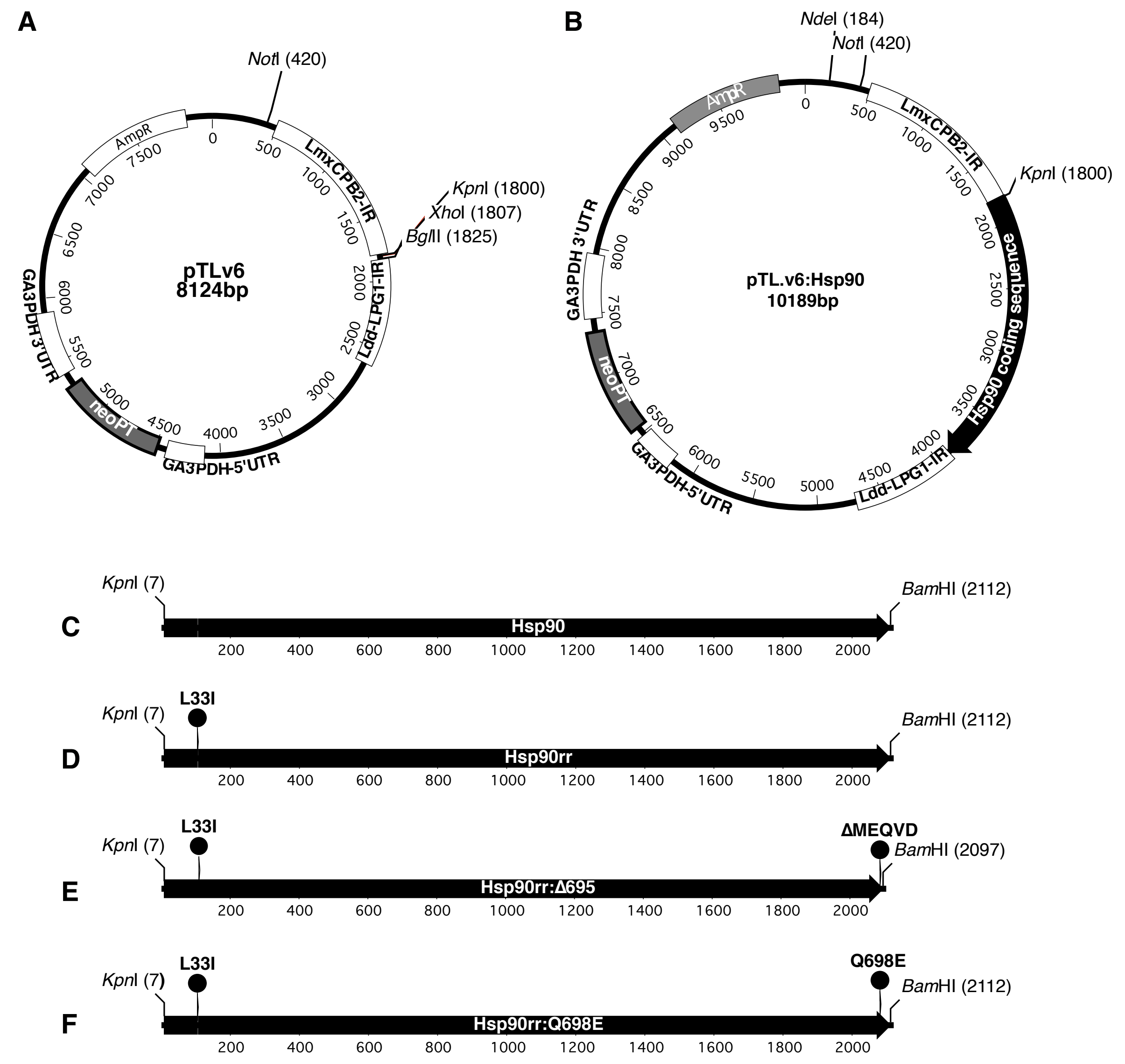

Schematic representation of *Hsp90* transgenes. The plasmid vector pTLv6 (A) was combined from the cosmid pcosTL (Kelly et al., 1994) and pIRmcs3+ (Hoyer et al., 2005) and allows the stable episomal establishment of transgenes in *Leishmania* spp. Coding sequences for Hsp90 variants were excised from pUC19 with *Kpn*I and *Bam*HI and fused between the *Kpn*I and *Bgl*II sites of pTLv6 (B). The Hsp90 variants are schematically drawn up with mutation sites highlighted (C-F).

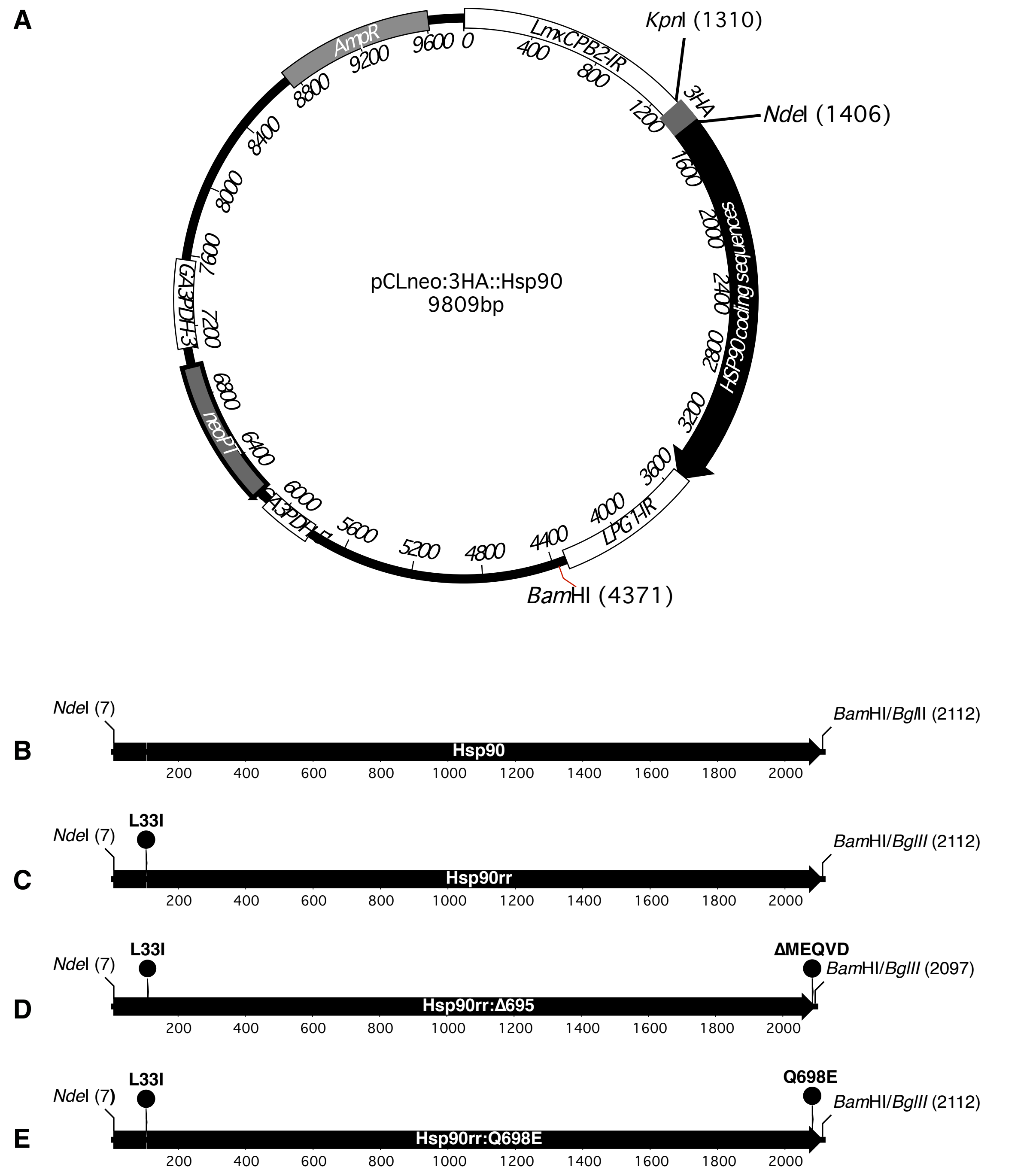

Schematic representation of 3xHA-tagged Hsp90 transgenes. The plasmid vector pCLneo:3HA (A) was opened with *Nde*I and *Bgl*II. The coding sequences for the Hsp90 variants were amplified by PCR to create *Nde*I and *Bam*HI sites, subcloned into pUC19 and verified, and then fused between the *Nde*I and *Bgl*II sites of pCLneo:3HA. The Hsp90 variants are schematically drawn up with mutation sites highlighted (B-E).

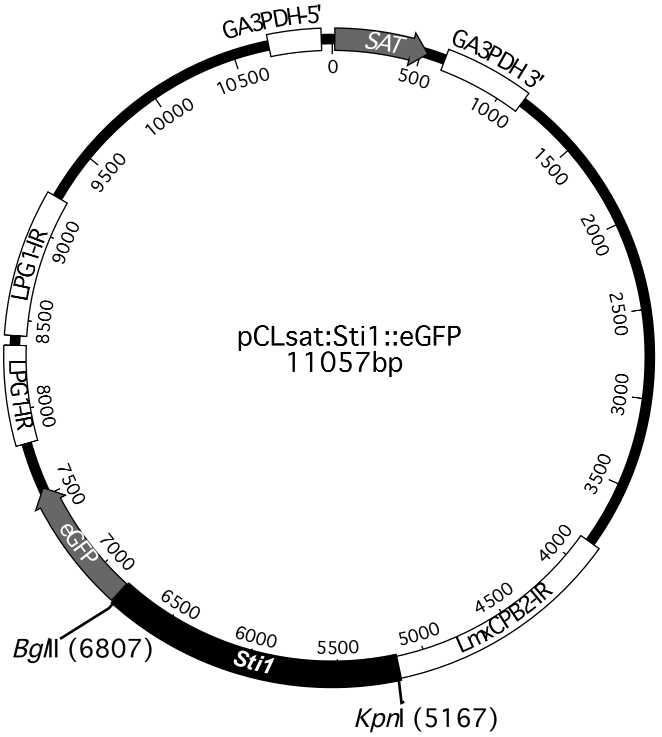

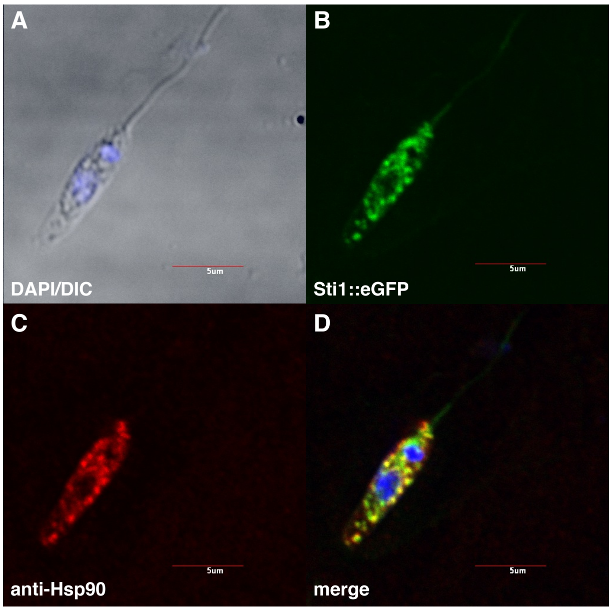

Upper panel: Schematic representation of pCLsat:Sti1::eGFP. The streptothricine (SAT) resistance marker expression is driven by *T. cruzi* glyceraldehyde 3' phosphate dehydrogenase gene flanking sequences. Sti1::eGFP fusion gene expression is under control of the *L. mexicana* cystein proteinase B2 intergenic region and the *L. donovani LPG1* 3' UTR.

Lower panel: Fluorescence microscopy imaging of Sti1::eGFP and anti-Hsp90 immune staining.

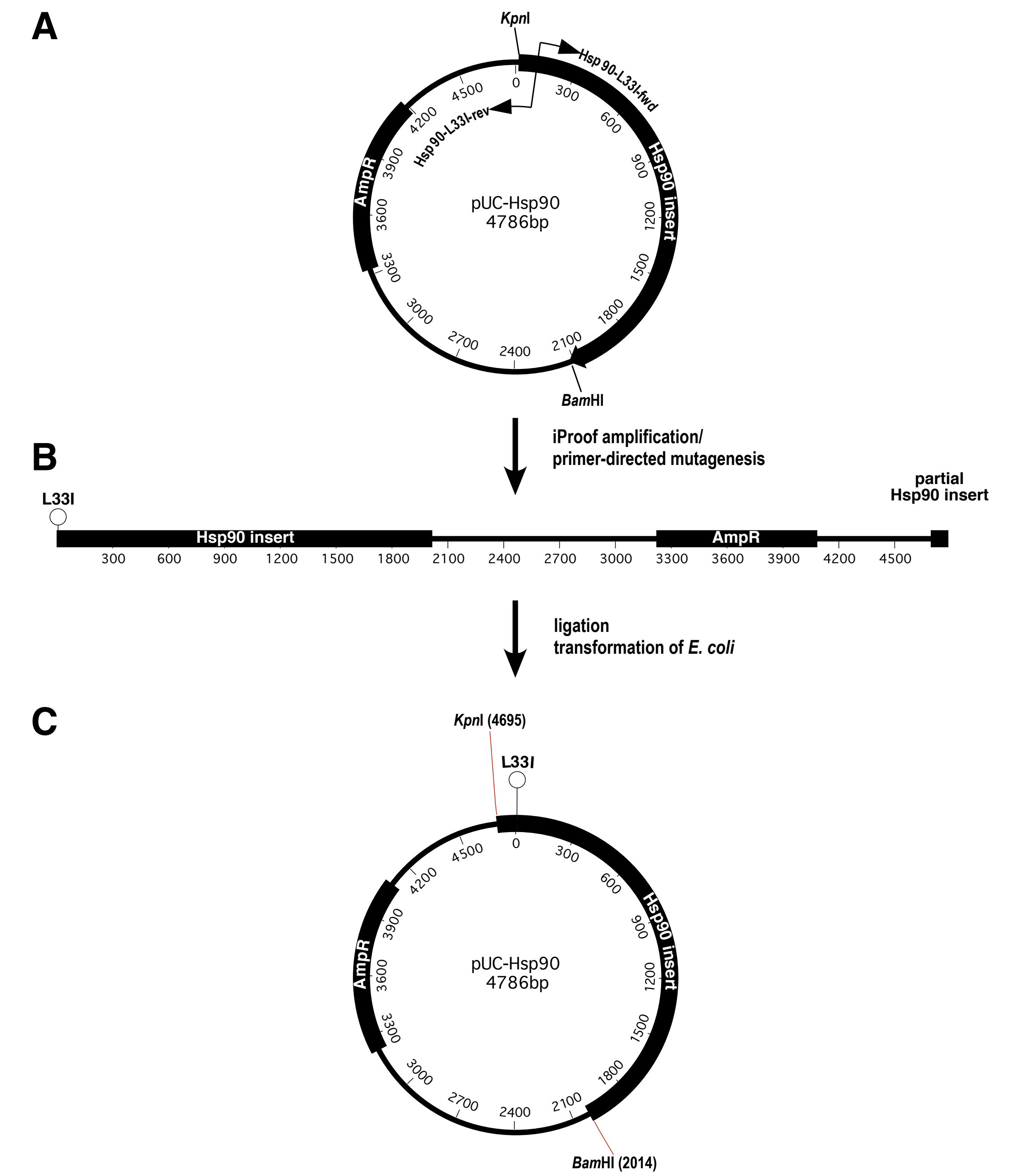

Schematic representation of primer directed mutagenesis. Hsp90 coding sequences were amplified and ligated into plasmid pUC19. A forward primer bearing the desired mutation 6 base pairs from its 5' end anneals immediately downstream from a reverse primer with wild type sequence (A). Both primers were 5'-phosphorylated prior to PCR using polynucleotide kinase. Amplification using the iProof kit with the buffer for G/C-rich DNA yielded a linear product (B) bearing the planned mutation. Ligation of the blunt ends then produced the pUC-Hsp90 plasmid with the mutation (C). Using *Kpn*I and *Bam*HI, the coding sequence was excised for insertion into the expression vector pTLv6 (SM3).
